# No vehicle, no problem

**DOI:** 10.18632/oncotarget.22100

**Published:** 2017-10-27

**Authors:** Esteban A. Orellana, Andrea L. Kasinski

**Affiliations:** Andrea L. Kasinski: Department of Biological Sciences, Purdue University, West Lafayette, IN, USA; Purdue Center for Cancer Research, Purdue University, West Lafayette, IN, USA

**Keywords:** miRNA, siRNA

In the last 8 years the field of microRNA (miRNA) replacement therapeutics has generated a lot of interest and at the same time skepticism due to failures. The therapeutic prospect is to use small synthetic RNA molecules that mimic endogenous miRNAs to restore the miRNA levels in cells that have lost them with the final goal of modulating cellular pathways that drive pathogenesis. However, as with other small RNA therapeutics (small interfering RNA, siRNA; antisense oligonucleotides), the progression of miRNAs into the clinic is hindered by the shortcomings of the available delivery systems. In a recent study, Orellana et al. showcases an approach that uses a therapeutically relevant miRNA mimic (miR-34a) that is directly conjugated to folate (FolamiR), the ligand of the folate receptor which is overexpressed in cancerous cells, to deliver functionally active miRNAs to cancer cells [1]. The overall postulate of this delivery strategy states that by removing the vehicle, unwanted toxicity could be reduced.

The experiments performed in this study show that the platform works both in cell-based studies using human triple negative breast cancer cells and *in vivo* in a human breast cancer model and in a murine model of lung adenocarcinoma. The data suggests that FolamiRs are selectively taken up by cells that overexpress the folate receptor, but not by normal cells and that the miRNA retains its activity and thus, is able to slow down cell and tumor growth. In addition, Folate-siRNA conjugates could also be delivered, arguing that the approach may be compatible with other types of small RNAs. These results are promising because the use of ‘naked’ miRNAs is not heavily pursued as a mean of systemic delivery due to the fact that these molecules are quickly excreted or degraded in serum, only achieve therapeutic levels in the liver and kidneys, and have been associated with activation of the immune system, leading to inflammatory responses [2]. For that reason, Orellana et al. attacked these concerns simultaneously by including minimal chemical modifications to the miRNA mimic backbone (2´ -O- Methyl modifications) and conjugated the mimic to a targeting ligand whose receptor is overexpressed in cancer cells, but is minimally expressed in normal cells [3]. The chemical modifications of the ribose backbone provided protection from nuclease degradation, and dampened immune stimulation. Interestingly, the covalent conjugation of the miRNA mimic to folate also provided additional protection and durability. For instance, unconjugated miRNA duplexes were quickly degraded in serum (1 hour) while FolamiR conjugates remained intact for more that 6 hours with signs of degradation present at 24 hours. It is unclear how folate protects the miRNA from degradation, but one possibility is that the folate moiety protects one of the ends of the RNA duplex from exonucleolitic cleavage. It would be interesting to investigate if conjugation to other ligands provides similar protection or if this phenomenon is unique to the folate ligand.

Lack of toxicity was confirmed at the highest dose used of 26.64 mg/kg; thus, providing support to the hypothesis that eliminating the vehicle could help to reduce toxicity without impairing delivery or miRNA activity. Furthermore, due to specific targeting and rapid clearance from the body it would be unlikely that the miRNA itself could reach toxic levels. Although the experiments have yet to reach humans, it is plausible that these molecules could help reduce unwanted toxicity along two lines simultaneously: specificity of uptake and rapid systemic clearance, both of which could be important to achieve reduced dosing in the absence of vehicle-associated toxicity to treat various diseases, including cancer. In the absence of toxicity, the limiting factor of dosage would likely be the internalization kinetics of the ligand bound to its receptor (endocytosis for folate - folate receptor) and the rate by which the FolamiR can escape the endosome. Endosomal sequestration is indeed an important limitation of ligand-mediated delivery. We anticipate that the development and inclusion of endosomal escape mechanisms into new generation FolamiRs could help overcome this hurdle, provide higher efficacy, and further reduce toxicity. We envision that for the case of cytostatic miRNAs (i.e. miR-34a) these FolamiRs could be combined with current chemotherapies to identify superior options to manage cancer and perhaps reduce chemoresistance. Another possibility would be conjugation of the targeting ligand to a cytotoxic small RNA to achieve tumor reduction. This delivery technology could also be used to deliver other types of small RNAs. One possibility, that has not yet been explored using ligands is the delivery of small guide RNAs (sgRNAs) that could be used for CRISPR-Cas9 gene editing.

Although these findings are encouraging, miRNA delivery has by no means been solved. Ligand mediated delivery has been used previously for siRNA therapies targeted to the liver by conjugating N-acetyl galactosamine (GalNAC), a ligand for the asialoglycoprotein receptor (ASGPR) present on hepatocytes [4]. Thus, arguing that perhaps this approach could be useful to overcome the shortcomings of current delivery technologies. We are currently pursuing the use of alternative ligand/receptor pairs to deliver miRNAs/siRNAs beyond cells that overexpress the folate receptor.

The challenge of the field is to continue to design and test different targeting ligands on miRNAs such that they can be delivered to the intended tissue quickly and with high specificity. As new delivery platforms are developed and our understanding of the safety profiles of miRNA mimics continues to be unraveled, the potential of transitioning relevant miRNAs into the clinic could become a not too distant possibility.

**Figure 1:**
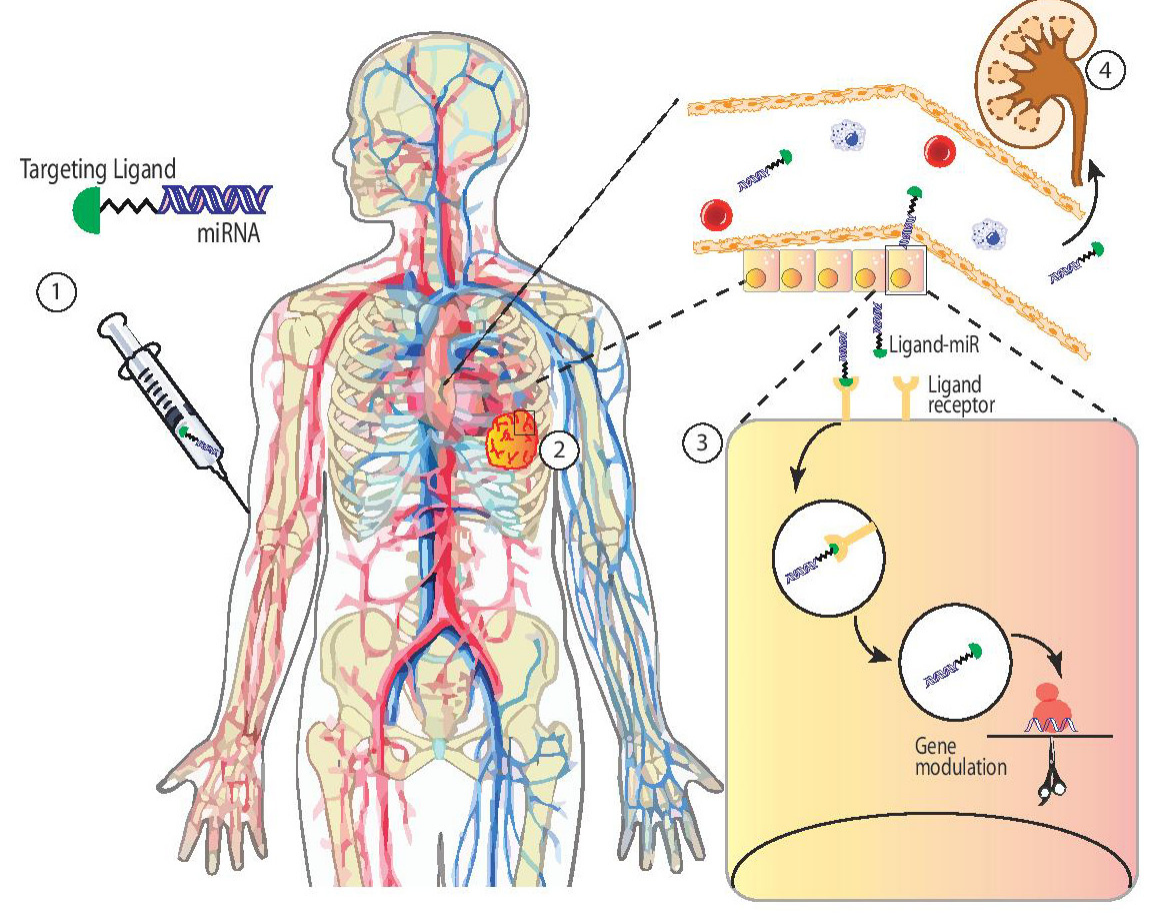
Ligand mediated miRNA replacement therapy Ligand-miRNAs enter circulation (1), localize to target tissue (2), and get internalized into target cells via ligand receptors (3). Excess ligand-RNA gets excreted from the body (4) avoiding unwanted toxicity.
